# Dust Aerosol Detection by the Modified CO_2_ Slicing Method

**DOI:** 10.3390/s19071615

**Published:** 2019-04-04

**Authors:** Yu Someya, Ryoichi Imasu, Kei Shiomi

**Affiliations:** 1 Center for Global Environmental Research, National Institute for Environmental Studies, Tsukuba 305-8506, Japan; 2 Atmosphere and Ocean Research Institute, The University of Tokyo, Kashiwa 277-8568, Japan; imasu@aori.u-tokyo.ac.jp; 3 Japan Aerospace Exploration Agency, Tsukuba 305-8505, Japan; shiomi.kei@jaxa.jp

**Keywords:** CO_2_ slicing method, dust aerosol, GOSAT, hyper-spectral sounding, thermal infrared

## Abstract

Dust aerosols, which have diverse and strong influences on the environment, must be monitored. Satellite data are effective for monitoring atmospheric conditions globally. In this work, the modified CO_2_ slicing method, a cloud detection technique using thermal infrared data from space, was applied to GOSAT data to detect the dust aerosol layer height. The results were compared using lidar measurements. Comparison of horizontal distributions found for northern Africa during summer revealed that both the relative frequencies of the low level aerosol layer from the slicing method and the dust frequencies of CALIPSO are high in northern coastal areas. Comparisons of detected layer top heights using collocated data with CALIPSO and ground-based lidar consistently showed high detection frequencies of the lower level aerosol layer, although the slicing method sometimes produces overestimates. This tendency is significant over land. The main causes of this tendency might be uncertainty of the surface skin temperature and a temperature inversion layer in the atmosphere. The results revealed that obtaining the detailed behavior of dust aerosols using the modified slicing method alone is difficult.

## 1. Introduction

Dust aerosols derive from dust storms in arid regions and subsequent widespread transport of their particles by winds. The Sahara and Sahel areas, located respectively from northern Africa to the Middle East and in central Asia from China to Mongolia on the subtropical high-pressure belt, are the major sources of such aerosols [1]. The main constituent species of dust aerosols are quartz, clays, calcite, gypsum, and iron oxides. Reportedly, the dust particles are not hygroscopic or moderately hygroscopic [2,3]. Dust aerosols from the Taklamakan and Gobi deserts are transported by westerly winds to eastern Asian nations such as China, Korea, and Japan, and even to North America [4,5,6]. Dust aerosols in those regions are called “yellow sand” or “Kosa”. These transported dust aerosols cause severe atmospheric pollution and influence social activities and human health in eastern Asia. Recently, the frequency of the dust events has reportedly increased because of changes in vegetation and precipitation around the source regions [7,8,9].

Transportation of dust aerosols has been studied widely using ground-based observations such as light detection and ranging (lidar), which can obtain vertical distributions of aerosols during daytime and nighttime, has been used since the end of the 1970s. To date, several observation campaigns or networks with ground-based lidar were implemented. For instance, the European Aerosol Research Lidar Network (EARLINET) [10,11] was a project for three years from 2000 to provide a quantitative and statistically relevant dataset of the vertical aerosol distribution over Europe. Such networks or observations at different sites have been useful to investigate how dust aerosols are transported and how their properties change [12,13,14,15]. These datasets are also used to validate transport model calculations [16]. Recent studies have used their vertical transportation to derive their vertical layering structure from dust aerosol measurements over the ocean [17,18].

Observations from space of dust storms in desert areas have been undertaken since the 1970s. Although several studies were conducted to detect dust aerosols using visible (VIS) and near-infrared (NIR) data ([19,20,21]), detecting them over desert surfaces is challenging because of the high reflectance. These techniques are applicable only over oceans or low-reflectance surfaces [22]. However, thermal infrared (TIR) observations are affected less by surface reflectance and are obtainable at night. In general, the existence of dust aerosols strongly affects remote sensing measurements not only in the VIS/NIR region but also in the TIR region because the particles are larger than those of aerosols of other types. Using brightness temperature differences, Shenk and Curran [23] and Ackerman [24] detected dust aerosols from TIR measurement data. Nevertheless, dust detection using TIR data presents difficulties that arise from surface temperature biases and small temperature contrasts between the ground surface and the aerosol layer. After multi-wavelengths measurements were enabled simultaneously, solutions to distinguish dust aerosols from the others were proposed. For instance, cirrus clouds are distinguished by brightness temperature differences between 11 μm and 12 μm, low clouds are distinguished by reflectance at 0.65–0.85 μm, and surfaces are distinguished by the ratio of reflectivity between 3.75 μm and 11 μm or brightness temperature differences between 11 μm and 12 μm. Large-scale dust storms are also detected from the signature at 1.6 μm [25]. After launching of hyper-spectral TIR sounders, spectral characteristics in the TIR window region of dust aerosols were elucidated [26,27,28]. Furthermore, optical thickness and layer height were retrieved from the sounding data [29,30,31,32,33,34]. High-resolution NIR spectra near oxygen-A band are also useful to derive dust layer height by application of the cloud detection algorithm to dust aerosols [35]. Since the launch of the space-borne lidar, Cloud–Aerosol Lidar with Orthogonal Polarization (CALIOP) on Cloud–Aerosol Lidar and Infrared Pathfinder Satellite Observation (CALIPSO) [36], aerosol’s vertical distribution have been obtainable globally [37,38,39]. Recently, CALIPSO data have come to be used to constraint aerosol transport models [40,41,42].

This study investigated the performance of the modified CO_2_ slicing method developed for cloud detection from Greenhouse Gases Observing Satellite (GOSAT) data [43] for application to dust aerosol detection. GOSAT cloud screening is based on an image sensor, Cloud and Aerosol Imager (CAI), which has four bands in the near-infrared and visible regions. However, using CAI for the screening of dusty scenes, especially over desert surfaces, is quite difficult for the same reasons as those applicable to VIS/NIR sensors described above. Moreover, information related to the cloud or aerosol layer height is unobtainable from CAI data. Contamination by clouds or aerosols during gas retrieval must be avoided. The most important objective of this study is to detect dusty scenes and to obtain their layer top height (LTH) or layer top pressure (LTP) to decrease contamination by dust aerosols during gas retrieval. Data used for this study and the analytical method are presented in Section 2. In Section 3, results from GOSAT data analyses are compared with measurements taken using space-borne and ground-based active instruments.

## 2. Data and Methods

### 2.1. GOSAT Data

A Japanese satellite, GOSAT, was launched in January 2009 to monitor carbon dioxide and methane concentrations. It is a Sun-synchronous polar orbit satellite at approximately 666 km height with a revisit cycle of three days and equator-crossing local time (LT) of 13:00. An instrument designated as Thermal And Near-infrared Sensor for Observation (TANSO) [44] on GOSAT consists of a Fourier transform spectrometer (FTS) and CAI. They have four bands in different spectral regions: FTS observes in the range of 0.758–0.775 μm (Band 1), 1.56–1.72 μm (Band 2), and 1.92–2.08 μm (Band 3) in the SWIR region and also 5.5–14.3 μm (Band 4) in the TIR region, with spectral resolution of about 0.2 cm^−1^; CAI observes at 0.380 μm (Band 1), 0.674 μm (Band 2), 0.870 μm (Band 3), and 1.60 μm (Band 4) with spatial resolution of 0.5 km (Bands 1–3) and 1.5 km (Band 4). The FTS’s size of the instantaneous field of view (IFOV) is 15.8 mrad, which corresponds to diameter of approximately 10.5 km at the Earth’s surface. TANSO-FTS has several observation patterns. The maximum pointing angle is ±35° in a cross-track direction and ±20° in an along-track direction [44]. In our study, TANSO-FTS Band 4 Level 1B (L1B) ver. 160.161 products (spectral radiance data) obtained during 2010–2012 provided by the National Institute of Environmental Studies (NIES) was used.

### 2.2. Lidar Data

CALIPSO data were used to validate aerosol layers retrieved from the GOSAT data. CALIOP is a lidar measurement system with laser wavelength of 532 nm and 1064 nm. It is currently the most accurate means of detecting aerosols from space. CALIPSO is also a sun-synchronous polar orbit satellite with a revisit cycle of 16 days; its equator-crossing time is 13:30 (local time). Vertical resolutions of sampling are 30 m below 8.2 km and 60 m between 8.2 km and 20.2 km. The lidar footprint is a circle with about 90 m diameter at the surface. The spatial interval of footprints is 333 m along a track. The CALIOP Level 2–5 km Aerosol/Cloud Layer V3.01 and V3.02 products were used for this study. These products include information related to aerosol layers such as the number of layers up to 10, geometrical layer top and bottom height, optical thickness, and particle species. Dust and polluted dust flags are included in the particle flag in the Level 2 (L2) aerosol products.

Ground-based lidar measurement data provided by the NIES lidar network (http://www-lidar.nies.go.jp) [45] are also used for validation. This network comprises two-wavelength (532 and 1064 nm) and polarized (532 nm) ground-based lidar at 13 sites in Japan, three sites in Korea, three sites in Mongolia, one site in Thailand, and four cooperative sites in China. Data from Japan, Korea, Mongolia, and Thailand are processed in real time and are available online. Backscatter coefficients up to 18 km, depolarization ratios up to 18 km, and extinction coefficients for spherical and dust particles up to 6 km are provided for every 15 min.

### 2.3. GOSAT Data Analysis Strategy

The GOSAT TIR L1B spectra were analyzed with the modified CO_2_ slicing algorithm for the operational processing of GOSAT-2 TIR L2 cloud/aerosol products described in an earlier report [43]. The CO_2_ slicing method uses the difference between the channel pair of the CO_2_ absorption strength near 14 μm [46,47,48] according to the equation:

(1) $$\frac{R_{\lambda_{1}}-R_{\lambda_{1}}^{clr}}{R_{\lambda_{2}}-R_{\lambda_{2}}^{clr}}=\frac{\alpha_{1}\epsilon_{\lambda_{1}}\int_{p_{s}}^{p_{c}}t_{\lambda_{1}}\left(p\right)dB_{\lambda_{1}}}{\alpha_{2}\epsilon_{\lambda_{2}}\int_{p_{s}}^{p_{c}}t_{\lambda_{2}}\left(p\right)dB_{\lambda_{2}}},$$

where R stands for the observed radiance, $R^{clr}$ denotes the calculated clear sky radiance, α signifies a cloud fraction in the IFOV, ϵ represents the cloud emissivity, $p_{s}$ and $p_{c}$ respectively denote pressure at the surface and the cloud top, t denotes the transmittance, B is the Planck function, and subscript λ denotes the spectral channel wavelength. With application to satellite data, t is calculated using LBLRTM at each layer level and $R^{clr}$ is calculated with the theoretical radiative transfer calculation from t, surface skin temperature, and surface emissivity based on GSM-GPV and ASTER database. If the two spectral channels $\lambda_{1}$ and $\lambda_{2}$ are sufficiently close, it can be assumed that the fractions and the emissivity are equal ($\alpha_{1}\epsilon_{1}≅\alpha_{2}\epsilon_{2}$). The value αϵ, called the Effective Cloud Amount (ECA), corresponds to the coverage if clouds in the IFOV are opaque or cloud emissivity if clouds are homogeneous in the IFOV. The LTP can be estimated from the calculations of this equation at each level of atmosphere. Estimated LTP can be converted to LTH with the assumption of hydrostatic equilibrium. The modified algorithm reconstructs pseudo-channels from the original channels based on the sensitivity height. The channel pair selection is optimized for several typical temperature profiles as indicators of latitude and temperature at 500 hPa. This algorithm does not use spectral dependency of the refractive index of dust particles but detects atmospheric absorbing matter using the narrow range of the TIR region. Therefore, dust particles are treated equivalently to cloud particles. The scene is regarded as clear if the lowest level of the vertical grid in the calculation is detected.

The Line-By-line Radiative Transfer Model [49] ver. 12.4 provided by Atmospheric Environmental Research Inc. was used for radiative transfer calculations considering gas absorption based on the high-resolution transmission molecular absorption database 2012 [50]. Atmospheric states and the surface temperature necessary for radiative transfer calculations were obtained from the Global Spectral Model-Grid Point Value (GSM-GPV) which are reanalysis data provided by the Japan Meteorological Agency. Over the ocean, sea surface temperature (SST) in GSM-GPV was used as the surface skin temperature. Over land, the surface skin temperature was assumed as equal to the surface air temperature because GSM-GPV includes no land surface skin temperature. The Thermal Emission and Reflection Radiometer Spectral Library [51] is based on the land cover type from the International Geosphere–Biosphere Programme.

## 3. Results

In two areas, Asia and the Sahara, dust storms are frequently observed. Results obtained from the slicing method were compared to those obtained from other measurements, CALIPSO, and ground-based lidar in these areas. In the Sahara area, large-scale dust storms occur widely: it is therefore easy to compare their horizontal distributions. Asian dust transported from inland areas of Asia is observed throughout eastern Asia in China, Korea, and Japan although the horizontal scales are slightly smaller than those of the Sahara. In Section 3.1, the results are compared with the CAI image and the ground-based lidar for the Asian dust events. In Section 3.2, they are compared with CALIPSO data obtained for northern Africa and the whole Earth.

### 3.1. Comparison in Eastern Asia

**Figure 1:**
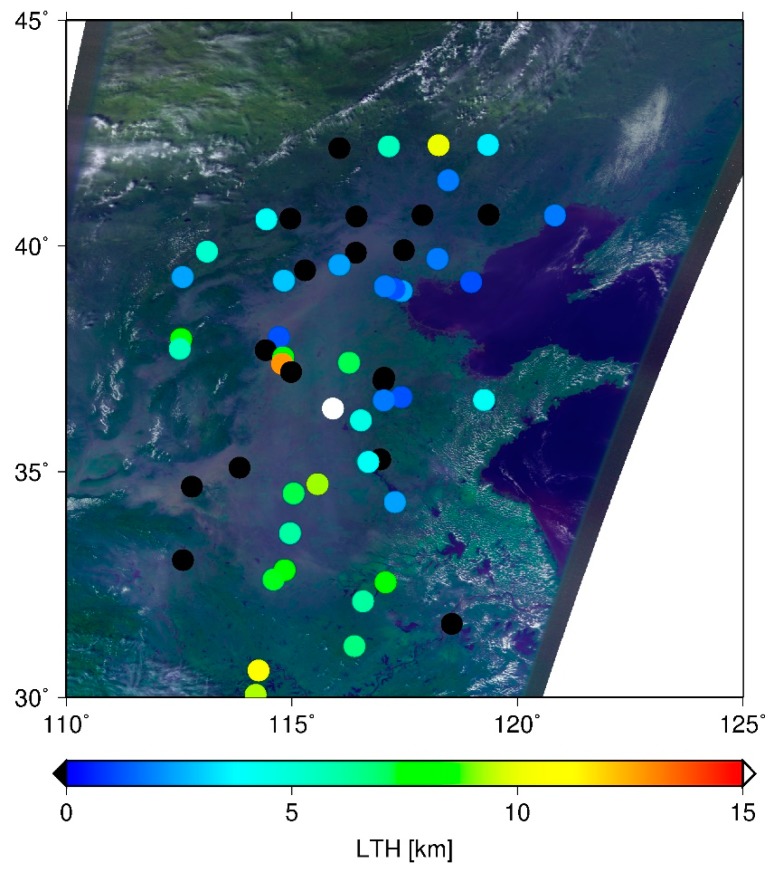
Distributions of the LTHs detected using the slicing method and the corresponding CAI image in eastern China on 9 October 2010. The LTHs detected by slicing are shown in color; black shows detection as clear.

Asian dust storms frequently occur in eastern Asia. Their sources are the Taklamakan and Gobi deserts. Dust plumes are transported by westerly winds to the countries of East Asia. Figure 1 presents an example of the distributions of LTHs detected using the slicing method and the corresponding CAI image during the dust event on 9 October 2010 in eastern China. The dust plume is readily apparent in the CAI image. Although the eastern part of the plume was detected using the slicing method, several points in the western part were inferred as clear sky. A possible cause is the difference of layer heights of particles. The light particles are blown up easily to the upper level and are transported faster in the downwind side. By contrast, heavy particles stay in the lower level or settle out by gravity on the upwind side. The slicing method is unable to detect the near surface level because, if the lowermost level in the calculation is detected as LTH, it is judged as clear sky. Therefore, the layer height in the western part might be too low to be detected using the slicing method.

**Figure 2:**
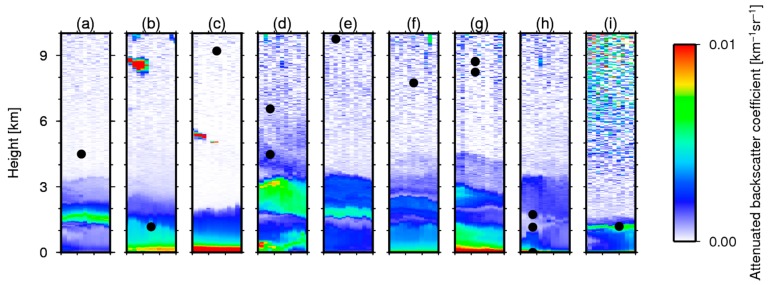
Temporal and vertical distribution of attenuated backscatter coefficient at 1064 nm from lidar observations and the detected layer height from the slicing method for the collocated data (black circle). Data were obtained (**a**) at Osaka on 14 March 2010, (**b**) on 20 March 2010, (**c**) on 2 May 2011, (**d**) at Chiba on 6 April 2010, (**e**) on 6 May 2010, (**f**) at Hedo 1 May 2010, (**g**) at Matsue on 4 May 2010, (**h**) at Tokyo on 6 April 2010, and (**i**) at Seoul on 17 January 2010. The *x*-axis ranges are 3:00 to 6:00 (UTC) for all cases.

The LTHs estimated using the slicing method were evaluated with ground-based lidar measurements from the NIES lidar network. The collocated GOSAT data are selected within 50 km between the center of the IFOV of TANSO-FTS/GOSAT and the lidar sites with the conditions that no clouds exist above the dust layer that has optical thickness greater than 0.05 and that the layer top is higher than 1 km. Actually, 31 such cases were identified for 2010–2012. Of them, the slicing method detected layers in 12 cases. All of these cases were in Japan. Figure 2 portrays the detected LTH from the slicing method and the attenuated backscatter coefficient at 1064 nm obtained from the collocated lidar measurements. Attenuated backscatter coefficient profiles obtained using the ground-based lidar are shown in color. The LTHs from the slicing method are displayed there as a black circle. More than one GOSAT measurement can correspond to one lidar measurement in some cases because of the match-up condition and the size of the IFOV of GOSAT. For all cases in the figure, dust layer tops were observed below 5 km from the lidar. However, the slicing method sometimes detects higher levels. In panels (b), and (i), the LTHs detected using the slicing method are close to those from the lidar measurements; in contrast, in panels (c), (d), (e), (f), and (g), the LTHs from the slicing method are overestimated.

### 3.2. Comparison of Global and Sahara Data

As explained in this section, results from the slicing method were compared with the CALIPSO measurements. The GOSAT data observed during three years of 2010–2012 and collocated with CALIPSO within 50 km horizontally and 15 min temporarily in the northern hemisphere were analyzed. The collocated data are only those of daytime because of their orbits. The CALIPSO measurements are selected by referring the product flags with the dust or polluted dust aerosol events without clouds above the aerosol layer. Table 1 presents the number of collocated unique dust or polluted dust layers detected by the CALIPSO 5 km averaging product and the detectability for those data by the slicing method, which is the ratio of the number of GOSAT data with detection of the layer for each surface type. It is noteworthy that several CALIPSO measurements can be matched up with one GOSAT measurement because the CALIPSO measurement interval along the track is narrower than that of GOSAT. In those cases, each CALIPSO measurement is counted. Actually, 32% of the dust events were detected using the slicing method. Over the ocean, 40% were detected, but to the figure is only 23% over land.

**Figure 3:**
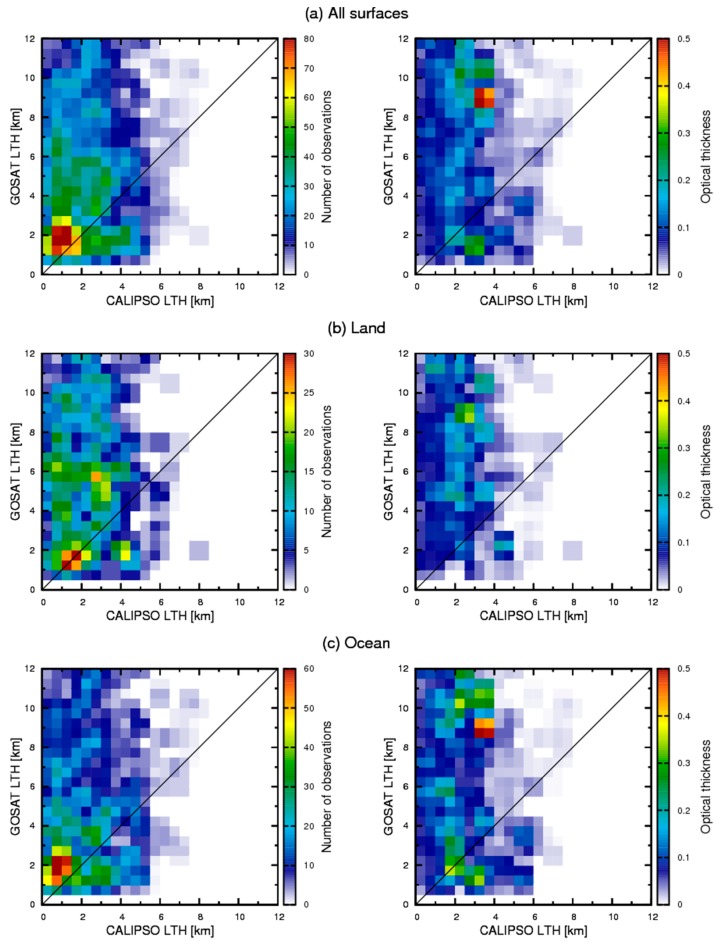
Left panels show the number density map of detected LTH between the slicing method and CALIPSO over (**a**) all, (**b**) land, and (**c**) ocean surfaces. Right panels show the averaged optical thickness of the aerosol layer from CALIPSO for each grid.

Left panels of Figure 3 present number density maps of detected LTHs for the slicing method and CALIPSO over (a) all, (b) land, and (c) ocean surfaces. The data are the same as those used in Table 1. The slicing method provided no detection at 0–0.5 km because it is regarded as clear if the lowermost layer was detected by the slicing method, as described. The maximum values are apparent in 1–3 km levels in both observations consistently. Although most LTHs of the CALIPSO observations were detected below 5 km, those of the slicing method were sometimes detected above 5 km. This tendency is stronger over land. Based on this fact, we infer that the slicing method tends to overestimate aerosol LTH and that the error is larger over land, probably because of uncertainty of the surface states such as temperature or emissivity, which is larger over land. Right panels of Figure 3 show the averaged optical thickness within each grid from CALIPSO. In the algorithm of the CALIPSO aerosol product, lidar ratios of 40 and 55 at 532 nm and 1064 nm are used for dust. For polluted dust, they are 65 and 30 respectively [37]. Over land, the slicing method overestimated LTH despite the large optical thickness. Moreover, there is small correlation between dust optical thickness and the detection accuracy of the slicing method. Over land, areas where optically thick dust aerosols are observed are arid regions. In these areas, the uncertainty of surface skin temperature is large, as discussed in Section 4. Therefore, it is possible that the error related to surface skin temperature is greater than the change of detectability depending on optical thickness. Over the ocean, peaks of optical thickness are seen at around 9 km and 1–2 km. The peak at the lower level is reasonable because large particles can exist at the lower level. For the peak at around 9 km, it is likely because of the inversion layer. The presence of the inversion layer can cause overestimation by the slicing method. This point is also discussed in Section 4.

**Figure 4:**
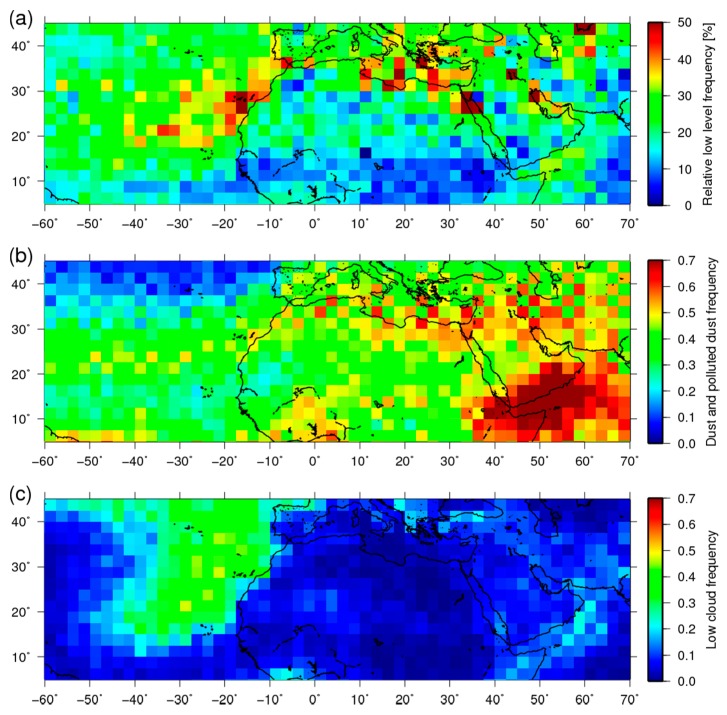
(**a**) Relative frequencies (scaled by the total frequency) of low level layer (≥660 hPa) obtained using the slicing method in northern Africa. (**b**) Dust and polluted dust frequencies of the CALIPSO aerosol product. (**c**) Low cloud frequency from the CALIPSO cloud product. The grid size is 2.5 × 2.5 deg.

Next, the horizontal distribution of detection frequencies of the slicing method is compared to that from CALIPSO around the Sahara area. Because the slicing method is unable to distinguish dust aerosols from clouds, the horizontal pattern of the detected layers is compared with those from the CALIPSO aerosol and cloud products in Sahara during summer, when dust events occur frequently. Figure 4 presents detection frequencies obtained using the slicing method and CALIPSO. Here, frequency means the ratio of the number of the detected observation to that of all observation within the grid. Figure 4a portrays the relative frequencies of the low-level layer (≥660 hPa), which are defined as the low-level frequency scaled by the total detection frequency from the slicing method. Figure 4b presents the dust and polluted dust frequencies by CALIPSO. Figure 4c depicts frequencies for low clouds (≥660 hPa) by CALIPSO in the boreal summer (June–August) during 2010–2012 within 2.5 × 2.5 degree grids. Using the slicing method, 40–50% of frequencies became visible in the northern and western coastal areas, whereas they are 20–30% in the other areas in panel (a). In the western coastal area, the frequencies of CALIPSO dust and low clouds are also high. Therefore, one can infer that the high frequencies of the slicing method include low clouds in this area. However, in the northern coastal area, the values of dust frequencies are large. Low cloud frequencies are small from CALIPSO. Therefore, the high frequencies of the slicing method in this area might be affected by dust aerosols. Over land, the frequencies of the slicing method are not so high, but those of dust from CALIPSO are high. This result is consistent with the tendency shown in Figure 3. The causes of this low detectability over land are discussed in Section 4. Dust events occur around the Arabian sea. Those are portrayed in Figure 4b. However, the frequencies in (a) are not so high. In this area, high clouds are also observed frequently by CALIPSO (not shown). Therefore, high clouds exist above the dust layer. In many cases, the slicing method was unable to detect the dust layer.

## 4. Discussion

An earlier section described that LTHs from the slicing method tend to be higher than those from CALIPSO and from the ground-based lidar, especially over land. Possible causes of this difference are the uncertainties of surface skin temperature, surface emissivity, temperature profile concentration of absorbing gas (CO_2_ in the used spectral region), and measurement accuracy of spectra. Dust aerosols are frequently observed over land are arid region. In these areas, CO_2_ emission and absorption are small. Therefore, model calculations assumed as the CO_2_ concentration in our analysis could consistent with real values. Temperature profiles were obtained from the reanalysis data, so it can also be reliable for the areas. Surface emissivity probably entails some uncertainty because some variation in the same surface type exists. Although all of them probably affected the results to some degree, surface skin temperature is possibly the largest error source in the results over land. That is true because the contribution from the uncertainty of surface skin temperature to the spectra can reach more than 5 K over land and those from the others are probably less than a few Kelvins. Lower level detection uses weak absorption bands. The contribution of radiation from the surface is large. This large uncertainty over land is attributable to the surface skin temperature, which is assumed to be equal to the surface air temperature for analysis over land, as described in Section 2. Nevertheless, the assumed surface skin temperature is probably lower than the real temperature in many cases because the collocated data were observed around 13:15 LT because of their orbit paths. Also, the surface skin temperature becomes much higher than the air temperature just above the surface because of solar heating. In these cases, the slicing technique overestimates LTH [43,52]. In addition, the difference between surface skin and air temperature depend on the land surface type because the variations of surface skin temperature are related with surface emissivity. Therefore, the accuracy of the modified slicing method also depends on that. Especially in desert areas where dust storms frequently occur, this overestimation can be noticeable because the difference is very large. This uncertainty of land surface skin temperature might also be the cause of the difference of detectability over land shown in Table 1. This might be a main cause of the low detectability over land in Figure 4.

Some overestimations of LTHs are also apparent over the ocean as shown in Figure 3. The slicing technique uses the temperature lapse rate of the atmosphere. Therefore, the inversion layers also cause error detection. In principle, the slicing can detect a higher level for a case in which the cloud or aerosol layer is in the inversion layer [43]. In western coastal areas of continents, inversion layers often occur because of low SST caused by upwelling flow. In the western coastal area of northern Africa, optically thick dust layer can occur with the inversion layer. It is explainable by the fact that the peak of optical thickness at 9 km of the right panel of Figure 3c.

The results of this study showed that the modified slicing method is not very effective for detecting dust aerosol layers. Although it appears that the behavior of dust aerosols can be slightly obtained using this method alone, the combinational use with other observations is probably more useful. The effectiveness of the slicing method is obtainable as the three-dimensional distributions of dust layer top albeit with large uncertainty. However, ground-based lidar can obtain vertical profiles at the site and space-borne imager can derive horizontal distributions. Therefore, the slicing method can provide additional information dimensionally. Moreover, it is likely to be able to constrain the spatial distributions of dust aerosols obtained from ground-based lidar or a space-borne imager.

## 5. Summary

The modified CO_2_ slicing technique was applied to three-year GOSAT data to evaluate the detectability of dust aerosols. The derived horizontal distribution and layer heights were validated through comparison with those from CALIPSO and ground-based lidar measurements. The slicing method detects 32% of dust events detected by CALIPSO for coincident observations completely. The detection was 23% over land and 40% over the ocean. Heights detected using the slicing method were consistently lower than 5 km with data obtained from CALIPSO, although those were overestimated in some cases, especially over land. This tendency was also apparent in the comparison with the ground-based lidar measurements. Main causes might be the uncertainty of surface skin temperature used for analyses over land or perhaps a temperature inversion layer over the ocean. The relative frequency of low-level detection from the slicing method and the dust frequency from CALIPSO showed large values around the northern coastal area of Africa. Although CO_2_ slicing method is widely used for cloud detection, its level of detectability for dust aerosols had not been evaluated. The advantage of this method is its ability to obtain the three-dimensional distributions of dust layers. However, this study revealed that the modified slicing method could not very effectively detect dust aerosols and their behaviors appear to be only slightly obtained using this method alone. The combinational use with other observations will probably provide more useful information.

## Figures and Tables

**Table 1:** Number of unique dust or polluted dust layers from CALIPSO collocated with GOSAT within 50 km/15 min for all (including mixed), land, ocean surfaces, with detectability defined by the ratio of the number of data for which dust layers are detected using the slicing method to the number of collocated CALIPSO measurement.

	All Surfaces	Land	Ocean
Number of Collocated Data	23,216	12,243	10,543
Detectability	0.32	0.23	0.40

## References

[1] Prospero J.M., Ginoux P., Torres O., Nicholson S.E., Gill T.E. (2002). Environmental characterization of global sources of atmospheric soil dust identified with the Nimbus 7 Total Ozone Mapping Spectrometer (TOMS) absorbing aerosol product. Rev. Geophys..

[2] Gustafsson R.J., Orlov A., Badger C.L., Griffiths P.T., Cox R.A., Lambert R.M. (2005). A comprehensive evaluation of water uptake on atmospherically relevant mineral surfaces: DRIFT spectroscopy, thermogravimetric analysis and aerosol growth measurements. Atmos. Chem. Phys..

[3] Kaaden N., Massling A., Schladitz A., MüLLER T., Kandler K., SchüTZ L., Weinzierl B., Petzold A., Tesche M., Leinert S. (2009). State of mixing, shape factor, number size distribution, and hygroscopic growth of the Saharan anthropogenic and mineral dust aerosol at Tinfou, Morocco. Tellus B Chem. Phys. Meteorol..

[4] Duce R.A., Unni C.K., Ray B.J., Prospero J.M., Merrill J.T. (1980). Long-range atmospheric transport of soil dust from Asia to the tropical North Pacific-temporal variability. Science.

[5] Uematsu M., Duce R.A., Prospero J.M., Chen L., Merrill J.T., McDonald R.L. (1983). Transport of mineral aerosol from Asia over the North Pacific-Ocean. J. Geophys. Res. Atmos..

[6] Husar R.B., Tratt D.M., Schichtel B.A., Falke S.R., Li F., Jaffe D., Gasso S., Gill T., Laulainen N.S., Lu F. (2001). Asian dust events of April 1998. J. Geophys. Res..

[7] Kurosaki Y., Mikami M. (2003). Recent frequent dust events and their relation to surface wind in East Asia. Geophys. Res. Lett..

[8] Kim J. (2008). Transport routes and source regions of Asian dust observed in Korea during the past 40 years (1965–2004). Atmos. Environ..

[9] Lee E.H., Sohn B.J. (2011). Recent increasing trend in dust frequency over Mongolia and Inner Mongolia regions and its association with climate and surface condition change. Atmos. Environ..

[10] Matthais V., Freudenthaler V., Amodeo A., Balin I., Balis D., Bösenberg J., Chaikovsky A., Chourdakis G., Comeron A., Delaval A. (2004). Aerosol lidar intercomparison in the framework of the EARLINET project. 1.Instruments. Appl. Opt..

[11] Böckmann C., Wandinger U., Ansmann A., Bösenberg J., Amiridis V., Boselli A., Delaval A., De Tomasi F., Frioud M., Grigorov I.V. (2004). Aerosol lidar intercomparison in the framework of the EARLINET project. 2.Aerosol backscatter algorithms. Appl. Opt..

[12] Papayannis A., Amiridis V., Mona L., Tsaknakis G., Balis D., Bösenberg J., Chaikovski A., De Tomasi F., Grigorov I., Mattis I. (2008). Systematic lidar observations of Saharan dust over Europe in the frame of EARLINET (2000–2002). J. Geophys. Res. Atmos..

[13] Ansmann A., Bösenberg J., Chaikovsky A., Comerón A., Eckhardt S., Eixmann R., Freudenthaler V., Ginoux P., Komguem L., Linné H. (2003). Long-range transport of Saharan dust to northern Europe: The 11–16 October 2001 outbreak observed with EARLINET. J. Geophys. Res. Atmos..

[14] Binietoglou I., Basart S., Alados-Arboledas L., Amiridis V., Argyrouli A., Baars H., Baldasano J.M., Balis D., Belegante L., Bravo-Aranda J.A. (2015). A methodology for investigating dust model performance using synergistic EARLINET/AERONET dust concentration retrievals. Atmos. Meas. Tech..

[15] Wiegner M., Groß S., Freudenthaler V., Schnell F., Gasteiger J. (2011). The May/June 2008 Saharan dust event over Munich: Intensive aerosol parameters from lidar measurements. J. Geophys. Res. Atmos..

[16] Müller D., Heinold B., Tesche M., Tegen I., Althausen D., Arboledas L.A., Amiridis V., Amodeo A., Ansmann A., Balis D. (2009). EARLINET observations of the 14–22-May long-range dust transport event during SAMUM 2006: Validation of results from dust transport modelling. Tellus B.

[17] Groß S., Gasteiger J., Freudenthaler V., Müller T., Sauer D., Toledano C., Ansmann A. (2016). Saharan dust contribution to the Caribbean summertime boundary layer—A lidar study during SALTRACE. Atmos. Chem. Phys..

[18] Rittmeister F., Ansmann A., Engelmann R., Skupin A., Baars H., Kanitz T., Kinne S. (2017). Profiling of Saharan dust from the Caribbean to western Africa—Part~1: Layering structures and optical properties from shipborne~polarization/Raman lidar observations. Atmos. Chem. Phys..

[19] Griggs M. (1975). Measurements of atmospheric aerosol optical thickness over water using erts-1 data. J. Air Pollut. Control Assoc..

[20] Carlson T.N. (1979). Atmospheric turbidity in Saharan dust outbreaks as determined by analyses of satellite brightness data. Mon. Weather Rev..

[21] Norton C.C., Mosher F.R., Hinton B., Martin D.W., Santek D., Kuhlow W. (1980). A model for calculating desert aerosol turbidity over the oceans from geostationary satellite data. J. Appl. Meteorol..

[22] King M.D., Kaufman Y.J., Tanre D., Nakajima T. (1999). Remote sensing of tropospheric aerosols from space: Past, present, and future. Bull. Am. Meteorol. Soc..

[23] Shenk W.E., Curran R.J. (1974). Detection of dust storms over land and water with satellite visible and infrared measurements. Mon. Weather Rev..

[24] Ackerman S.A. (1989). Using the radiative temperature difference at 3.7 and 11 μm to track dust outbreaks. Remote Sens. Environ..

[25] Shao Y., Dong C.H. (2006). A review on East Asian dust storm climate, modelling and monitoring. Glob. Planet. Chang..

[26] Sokolik I.N. (2002). The spectral radiative signature of wind-blown mineral dust: Implications for remote sensing in the thermal IR region. Geophys. Res. Lett..

[27] DeSouza-Machado S.G., Strow L.L., Hannon S.E., Motteler H.E. (2006). Infrared dust spectral signatures from AIRS. Geophys. Res. Lett..

[28] Han H.J., Sohn B.J., Huang H.L., Weisz E., Saunders R., Takamura T. (2012). An improved radiance simulation for hyperspectral infrared remote sensing of Asian dust. J. Geophys. Res..

[29] Pierangelo C., Chedin A., Heilliette S., Jacquinet-Husson N., Armante R. (2004). Dust altitude and infrared optical depth from AIRS. Atmos. Chem. Phys..

[30] DeSouza-Machado S.G., Strow L.L., Imbiriba B., McCann K., Hoff R.M., Hannon S.E., Martins J.V., Tanre D., Deuze J.L., Ducos F. (2010). Infrared retrievals of dust using AIRS: Comparisons of optical depths and heights derived for a North African dust storm to other collocated EOS A-Train and surface observations. J. Geophys. Res..

[31] Yao Z.G., Li J., Han H.J., Huang A.L., Sohn B.J., Zhang P. (2012). Asian dust height and infrared optical depth retrievals over land from hyperspectral longwave infrared radiances. J. Geophys. Res..

[32] Han H.-J., Sohn B.J. (2013). Retrieving Asian dust AOT and height from hyperspectral sounder measurements: An artificial neural network approach. J. Geophys. Res..

[33] Vandenbussche S., Kochenova S., Vandaele A.C., Kumps N., Mazière M. (2013). De Retrieval of desert dust aerosol vertical profiles from IASI measurements in the TIR atmospheric window. Atmos. Meas. Tech..

[34] Cuesta J., Eremenko M., Flamant C., Dufour G., Laurent B., Bergametti G., Höpfner M., Orphal J., Zhou D. (2015). Three-dimensional distribution of a major desert dust outbreak over East Asia in March 2008 derived from IASI satellite observations. J. Geophys. Res. Atmos..

[35] Kokhanovsky A.A., Rozanov V.V. (2010). The determination of dust cloud altitudes from a satellite using hyperspectral measurements in the gaseous absorption band. Int. J. Remote Sens..

[36] Winker D.M., Hunt W.H., McGill M.J. (2007). Initial performance assessment of CALIOP. Geophys. Res. Lett..

[37] Omar A.H., Winker D.M., Vaughan M.A., Hu Y., Trepte C.R., Ferrare R.A., Lee K.-P., Hostetler C.A., Kittaka C., Rogers R.R. (2009). The CALIPSO automated aerosol classification and lidar ratio selection algorithm. J. Atmos. Ocean. Technol..

[38] Winker D.M., Tackett J.L., Getzewich B.J., Liu Z., Vaughan M.A., Rogers R.R. (2013). The global 3-D distribution of tropospheric aerosols as characterized by CALIOP. Atmos. Chem. Phys..

[39] Marinou E., Amiridis V., Binietoglou I., Tsikerdekis A., Solomos S., Proestakis E., Konsta D., Papagiannopoulos N., Tsekeri A., Vlastou G. (2017). Three-dimensional evolution of Saharan dust transport towards Europe based on a 9-year EARLINET-optimized CALIPSO dataset. Atmos. Chem. Phys..

[40] Eguchi K., Uno I., Yumimoto K., Takemura T., Shimizu A., Sugimoto N., Liu Z. (2009). Trans-pacific dust transport: Integrated analysis of NASA/CALIPSO and a global aerosol transport model. Atmos. Chem. Phys..

[41] Yumimoto K., Eguchi K., Uno I., Takemura T., Liu Z., Shimizu A., Sugimoto N. (2009). An elevated large-scale dust veil from the Taklimakan Desert: Intercontinental transport and three-dimensional structure as captured by CALIPSO and regional and global models. Atmos. Chem. Phys..

[42] Georgoulias A.K., Tsikerdekis A., Amiridis V., Marinou E., Benedetti A., Zanis P., Alexandri G., Mona L., Kourtidis K.A., Lelieveld J. (2018). A 3-D evaluation of the MACC reanalysis dust product over Europe, northern Africa and Middle East using CALIOP/CALIPSO dust satellite observations. Atmos. Chem. Phys..

[43] Someya Y., Imasu R., Saitoh N., Ota Y., Shiomi K. (2016). A development of cloud top height retrieval using thermal infrared spectra observed with GOSAT and comparison with CALIPSO data. Atmos. Meas. Tech..

[44] Kuze A., Suto H., Nakajima M., Hamazaki T. (2009). Thermal and near infrared sensor for carbon observation Fourier-transform spectrometer on the Greenhouse Gases Observing Satellite for greenhouse gases monitoring. Appl. Opt..

[45] Sugimoto N., Matsui I., Shimizu A., Nishizawa T., Hara Y., Uno I. (2010). Lidar network observation of tropospheric aerosols. Proc. SPIE.

[46] Chahine M.T. (1974). Remote sounding of cloudy atmospheres. 1. single cloud layer. J. Atmos. Sci..

[47] Smith W.L., Platt C.M.R. (1978). Comparison of satellite-deduced cloud heights with indications from radiosonde and ground-based laser measurements. J. Appl. Meteorol..

[48] Menzel W.P., Smith W.L., Stewart T.R. (1983). Improved cloud motion wind vector and altitude assignment using VAS. J. Clim. Appl. Meteorol..

[49] Clough S.A., Shephard M.W., Mlawer E., Delamere J.S., Iacono M., Cady-Pereira K., Boukabara S., Brown P.D. (2005). Atmospheric radiative transfer modeling: A summary of the AER codes. J. Quant. Spectrosc. Radiat. Transf..

[50] Rothman L.S., Gordon I.E., Babikov Y., Barbe A., Chris Benner D., Bernath P.F., Birk M., Bizzocchi L., Boudon V., Brown L.R. (2013). The HITRAN2012 molecular spectroscopic database. J. Quant. Spectrosc. Radiat. Transf..

[51] Baldridge A.M., Hook S.J., Grove C.I., Rivera G. (2009). The ASTER spectral library version 2.0. Remote Sens. Environ..

[52] Menzel W.P., Wylie D.P., Strabala K.I. (1992). Seasonal and diurnal changes in cirrus clouds as seen in 4 years of observations with the VAS. J. Appl. Meteorol..

